# The causal association between smoking, alcohol consumption and risk of upper urinary calculi: insights from a Mendelian randomization study

**DOI:** 10.3389/fgene.2023.1268720

**Published:** 2023-11-30

**Authors:** Li Wang, Shan Yin, Kun-peng Li, Er-hao Bao, Jia-hao Wang, Ping-yu Zhu

**Affiliations:** ^1^ Department of Urology, Affiliated Hospital of North Sichuan Medical College, Nanchong, China; ^2^ Department of Urology, The Second Hospital of Lanzhou University, Lanzhou, China

**Keywords:** Mendelian randomization, upper urinary calculi, smoking, alcohol, risk

## Abstract

**Background:** The causal link between smoking, alcohol consumption, and upper urinary calculi remains uncertain in observational studies due to confounding factors. To uncover potential causal associations, we utilized two-sample univariable and multivariable Mendelian randomization (MR) methods.

**Methods:** Five risk factors related to lifestyles (cigarettes per day, lifetime smoking index, smoking initiation, drinks per week and alcohol intake frequency) were chosen from the Genome-Wide Association Study (GWAS). Upper urinary calculi were obtained from the FinnGen and United Kingdom Biobank consortium. Inverse-variance-weighted (IVW) was mainly used to compute odds ratios (OR) and 95% confidence intervals (Cl). While diligently scrutinizing potential sources of heterogeneity and horizontal pleiotropy via the rigorous utilization of Cochran’s Q test, the MR-PRESSO method, and MR-Egger.

**Results:** The summary OR for upper urinary calculi was 0.6 (IVW 95% CI: 0.49–0.74; *p* = 1.31 × 10^−06^) per standard deviation decrease in drinks per week. Interestingly, the genetically predicted alcohol intake frequency was associated with a significantly increased risk upper urinary calculi (OR = 1.27; 95% CI: 1.11–1.45; *p* = 0.0005). Our study found no association between smoking initiation, the number of cigarettes per day, and the lifetime smoking index and the risk of upper urinary calculi. By adjusting for body mass index and education, estimates of drinks per week remained consistent in multivariate MR analyses, while alcohol intake frequency became non-significant.

**Conclusion:** MR analysis showed that drinks per week was negatively associated with upper urinary calculi, whereas the effect of tobacco on upper urinary calculi was not significant and the detrimental effect of alcohol intake frequency on upper urinary calculi became non-significant after adjusting for BMI and education.

## 1 Introduction

There was a notable surge of 48.57% in the prevalence of urinary stones from 1999 to 2019, predominantly affecting the upper urinary tract-specifically, the kidneys and ureters (Zhu et al., 2021). These upper urinary calculi are highly prevalent and frequently result in debilitating conditions such as renal colic, hydronephrosis, and in severe cases, uremia (Hájková et al., 2021). Regrettably, the impact on patients’ quality of life is significant, with the lifetime incidence of kidney stones standing at 14% (Marić et al., 2019). More concerning is that at least half of those affected experience recurrent stone episodes within 10 years, leading to a substantial economic and lifestyle burden (Zhang et al., 2022).

Smoking and alcohol consumption are recognized as modifiable health behaviors with potential as risk factors. Extensive research has investigated their association with urolithiasis. yet the relationship between smoking, alcohol consumption, and the risk of urinary calculi remains uncertain, exhibiting conflicting findings in the epidemiological literature (Hall et al., 2001; Liu et al., 2009; Słojewski et al., 2009; Zhao et al., 2015). Several cross-sectional studies have posited an independent role for smoking in the formation of kidney stone (Hamano et al., 2005; Liu et al., 2009), whereas another study found that the quantity and duration of smoking were not significantly associated with stone formation (Słojewski et al., 2009; Marić et al., 2019). Moreover, a meta-analysis encompassing fourteen studies reported no significant impact of alcohol intake on the incidence of kidney stones (Jones et al., 2021). In contrast, a recent cohort study indicated a notable negative association between alcohol consumption and the formation of kidney stones (Kim et al., 2022).

The establishment of causality is crucial in clinical intervention planning and the formulating of public health policies. However, observational studies often grapple with confounding factors and the risk of reverse causality bias. While randomized controlled trials (RCTs) are the gold standard for elucidating etiological relationships, they are not without limitations in design and ethical constraints. Mendelian randomization (MR) offers a solution by using single nucleotide polymorphisms (SNPs) as instrumental variables (IVs) to ascertain causality, thereby reducing bias from confounding variables (Burgess and Labrecque, 2018). Multivariate Mendelian randomization (MVMR) extends the principles of univariate MR (UVMR) by accounting for the complexity of exposure characteristics and allows for simultaneous assessment of multiple, interrelated exposures (Sanderson, 2021).

In our study, we employed both UVMR and MVMR analyses on two separate cohorts to explore the potential causal link between genetic predisposition to smoking and alcohol consumption and the susceptibility to upper urinary calculi.

## 2 Methods

### 2.1 Study design

**FIGURE 1:**
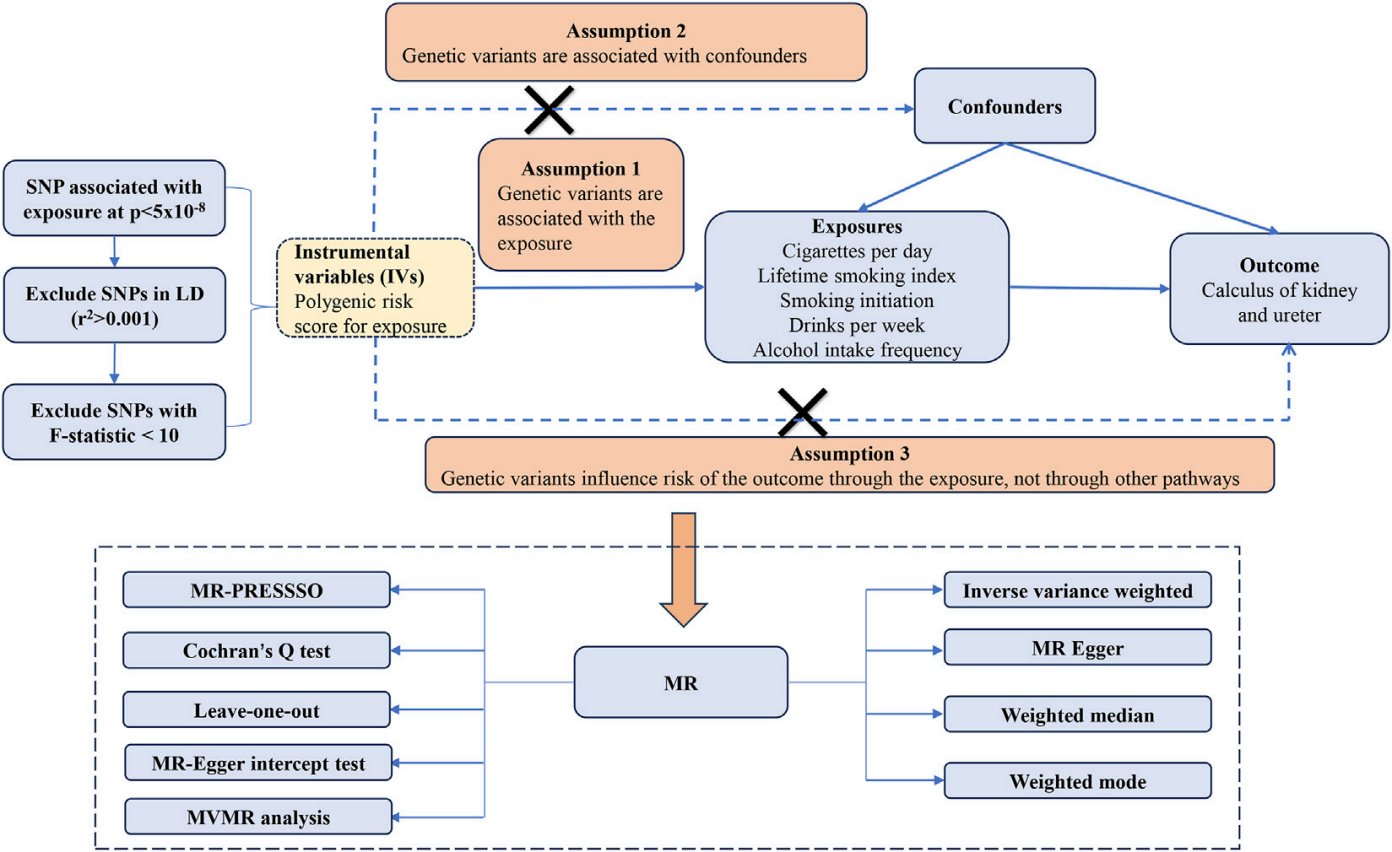
Flowchart of a MR study.

The study adhered rigorously to the guidelines set forth in the Strengthening the Reporting of Observational Studies in Epidemiology Mendelian randomization (STROBE-MR) framework (Supplementary Table S1) (Skrivankova et al., 2021). MR is predicated on three fundamental assumptions: IVs must exhibit a robust association with exposure, they should be unaffected by confounding variables, and they should influence the outcome exclusively through the exposure (Burgess et al., 2015). Our research implemented MR analyses in strict accordance with these core principles, as illustrated in Figure 1.

### 2.2 Choosing instrumental variables

To ensure the stability of the causal relationship between exposure and outcome, IVs were selected based on the following principles (Zhu et al., 2021): We established genome-wide significance thresholds for exposure at *p* < 5 × 10^−8^ (Hájková et al., 2021). Cluster analysis was conducted to address linkage disequilibrium (LD) among the selected IVs (*r* ^2^ < 0.001, kb = 10,000) (Marić et al., 2019). To mitigate bias from weak IVs. The strength of the IVs was quantified using the F value (β^2^/SE), with those having F < 10 being excluded. Here, β represents the effect size of exposure and SE represents the standard error of the effect size.

### 2.3 Data sources

Genetic summary data on smoking initiation, cigarettes per day, and alcohol consumption measured in drinks per week were acquired from the Sequencing Consortium of Alcohol and Nicotine use (GSCAN) (Saunders et al., 2022). This consortium’s dataset encompasses information from 2,669,029 individuals of European descent. The genome-wide association study (GWAS) dataset on the lifetime smoking index included details on smoking duration, intensity, and quitting, which were combined to create a simulated half-life (τ) constant and a lifetime smoking index (n = 462,690) (Wootton et al., 2020). GWAS data on the frequency of alcohol intake were obtained from the questionnaire “How often do you drink alcohol?” with ordered categorical variables (n = 462,346) (Hemani et al., 2018).

Genetic association summary data for upper urinary calculi were obtained from the ninth release of the FinnGen Consortium database, encompassing 376,406 individuals of Finnish ancestry, including both males and females (Kurki et al., 2023). The analysis excluded individuals exhibiting extreme heterozygosity (±4 SD), a genotyping deletion rate (>5%), ambiguous gender, or non-Finnish ancestry. The genetic associations’ effect sizes were estimated using logistic regression, controlling for age, sex, and principal genetic components. Additionally, cases of upper urinary calculi in the United Kingdom Biobank were identified based on the International Classification of Diseases, Ninth Revision (ICD-9) and 10th Revision (ICD-10) criteria. Correlation tests were adjusted for confounding variables, including age at enrolment, gender, and the first ten principal genetic components. Table 1 shows detailed information about the GWAS data.

**TABLE 1:** Phenotypic descriptive statistics of studies included in the exposure and outcome genome-wide association study.

Exposures/outcome	Type	Sample size N)	Consortium	Year	PubMed ID	Source
Smoking initiation	Categorical	3,383,199	GSCAN	2022	36,477,530	https://genome.psych.umn.edu/index.php/GSCAN
Cigarettes per day	Continuous	784,353	GSCAN	2022	36,477,530	https://genome.psych.umn.edu/index.php/GSCAN
Lifetime smoking index	Continuous	462,690	NA	2020	31,689,377	NA
Drinks per week	Continuous	2,965,643	GSCAN	2022	36,477,530	https://genome.psych.umn.edu/index.php/GSCAN
Alcohol intake frequency	Continuous	462,346	MRC-IEU	2018	NA	https://gwas.mrcieu.ac.uk/datasets/ukb-b-5779/
Calculus of kidney and ureter	Categorical	9,713/366,693	FinnGen	2022	NA	https://r9.finngen.fi/
Calculus of kidney and ureter	Categorical	5,530/420,531	United Kingdom Biobank	2022	NA	https://www.ukbiobank.ac.uk

GSCAN GWAS, and Sequencing Consortium of Alcohol and Nicotine use.

### 2.4 Power calculations

We conducted an *a priori* power calculation with an α of 5% (Brion et al., 2013). Ensuring that we had at least 80% power to detect Odds Ratios (ORs) for upper urinary calculi of 1.30, 1.42, 1.27, 1.25, and 1.10 for the respective variables of smoking initiation, cigarettes per day, lifetime smoking index, drinks per week, and alcohol intake frequency in the FinnGen data. Similarly, in the United Kingdom Biobank data, we could detect ORs of 1.11, 1.09, 1.51, 1.32, and 1.41 for these variables.

### 2.5 Other factors

To mitigate potential pleiotropy arising from indirect pathways, we utilized MVMR analyses. By consulting the PhenoScanner database, we pinpointed associations of IVs with education and obesity-related traits that met the genome-wide significance threshold (*p* < 5 × 10^−8^). Consequently, we meticulously selected SNPs linked to education (n = 1,131,881) (Lee et al., 2018) and BMI (n = 681,275) (Yengo et al., 2018) for our multivariate analysis.

### 2.6 Statistical analyses

Our primary analysis utilized the robust inverse-variance weighted (IVW) method (Burgess et al., 2013). For validation, we applied supplementary methods including weighted median, MR-Egger regression, and weighted mode. The weighted median approach, renowned for its reliability, provided consistent results by prioritizing the influence of the most powerful instrumental variable, which carried a 50% weight (Bowden et al., 2016). To tackle potential directional pleiotropy, we further employed MR-Egger regression and weighted mode methods (Bowden et al., 2015; Hartwig et al., 2017).

We assessed the genetic correlation for upper urinary calculi between the United Kingdom Biobank and FinnGen consortium using LD Score Regression (LDSC) software, which revealed high consistency (rg = 0.80; *p* = 3.75 × 10^−22^) (Bulik-Sullivan BK. et al., 2015; Bulik-Sullivan B. et al., 2015). Thus, we integrated the data using a fixed-effects model. I^2^ > 50% was interpreted as indicative of high heterogeneity.

Sensitivity analysis plays a critical role in evaluating heterogeneity and potential biases in MR studies. We first assessed heterogeneity using Cochran’s Q test, which calculates the weighted sum of squared differences between individual study estimates and the overall IVW estimate (Burgess et al., 2017). To detect and adjust for potential outliers, we applied the MR Pleiotropy RESidual Sum and Outlier (MR-PRESSO) detection method (Verbanck et al., 2018). Additionally, MR-Egger regression was utilized to test for potential horizontal pleiotropy by examining the regression intercept. Furthermore, the Steiger test was employed to rule out potential inverse associations (Hemani et al., 2017).

Statistical analyses were conducted utilizing R version 4.2.2, employing the “TwoSampleMR”, “MRPRESSO”, “meta” and “MVMR” packages. Odds ratios (ORs) with corresponding 95% confidence intervals (CIs) quantified the MR analysis, with a *p* < 0.05 denoting statistical significance.

## 3 Results

### 3.1 Selection of genetic variants and F-statistics

After the initial screening of SNPs, and subsequent removal of variants in potential linkage disequilibrium (threshold: *r* ^2^ = 0.001, 10,000 kb) and applying Steiger filtering, a total of 433, 84, 117, 232, and 90 SNPs were used as IVs for the number of smoking initiations, cigarettes per day, lifetime smoking index, drinks per week, and alcohol intake frequency, respectively. These SNPs explained 1.5%, 1.27%, 1.05%, 0.83%, and 2.13% of the phenotypical variance, respectively. Importantly, all the included SNPs had F-values exceeding 10, indicating a minimal likelihood of weak IVs bias (Supplementary Tables S2–S11).

### 3.2 Heterogeneity and pleiotropy

The absence of heterogeneity and the absence of directional pleiotropy were demonstrated in all analyses, as shown in Table 2. Furthermore, the MR-PRESSO analyses did not identify any outliers, indicating a robust dataset (all p for Global test >0.05) (Table 3).

**TABLE 2:** Heterogeneity and MR-Egger test for directional pleiotropy.

Exposure/Data source	Heterogeneity (IVW)		MR-Egger test for directional pleiotropy		
	Q	*P*	Intercept	Standard error	*P*
**Cigarettes initiation**					
FinnGen	23.5	0.234	−0.015	0.039	0.632
United Kingdom Biobank	18.7	0.632	0.003	0.022	0.745
**Cigarettes per day**					
FinnGen	104.4	0.952	0.004	0.006	0.503
United Kingdom Biobank	125.2	0.567	0.001	0.009	0.956
**Lifetime smoking index**					
FinnGen	132.6	0.124	−0.002	0.008	0.771
United Kingdom Biobank	150.1	0.062	−0.010	0.012	0.416
**Drinks per week**					
FinnGen	35.2	0.598	0.015	0.012	0.227
United Kingdom Biobank	41.2	0.331	−0.003	0.025	0.913
**Alcohol intake frequency**					
FinnGen	74.9	0.749	0.005	0.005	0.337
United Kingdom Biobank	108.8	0.075	0.004	0.006	0.438

**TABLE 3:** MR-PRESSO results.

Exposure/Data source	MR-PRESSO				
	OR	95% CI	*P*	RSSobs	*p*-value for global test
**Cigarettes initiation**					
FinnGen	0.87	0.72–1.06	0.186	522.8	0.225
United Kingdom Biobank	1.38	1.10–1.74	0.005	484	0.053
**Cigarettes per day**					
FinnGen	0.99	0.76–1.28	0.94	107.2	0.559
United Kingdom Biobank	0.79	0.59–1.06	0.13	127	0.959
**Lifetime smoking index**					
FinnGen	1	0.80–1.26	0.95	139.1	0.052
United Kingdom Biobank	1.74	1.31–2.30	0.0001	134.8	0.143
**Drinks per week**					
FinnGen	0.55	0.42–0.72	1.92E-05	209.5	0.332
United Kingdom Biobank	0.68	0.49–0.94	0.02	270.3	0.06
**Alcohol intake frequency**					
FinnGen	1.30	1.09–1.55	0.003	122.5	0.303
United Kingdom Biobank	1.21	0.98–1.49	0.06	110.8	0.07

OR, odds ratio; RSSobs, observed residual sum of squares.

### 3.3 Univariate MR analysis

**FIGURE 2:**
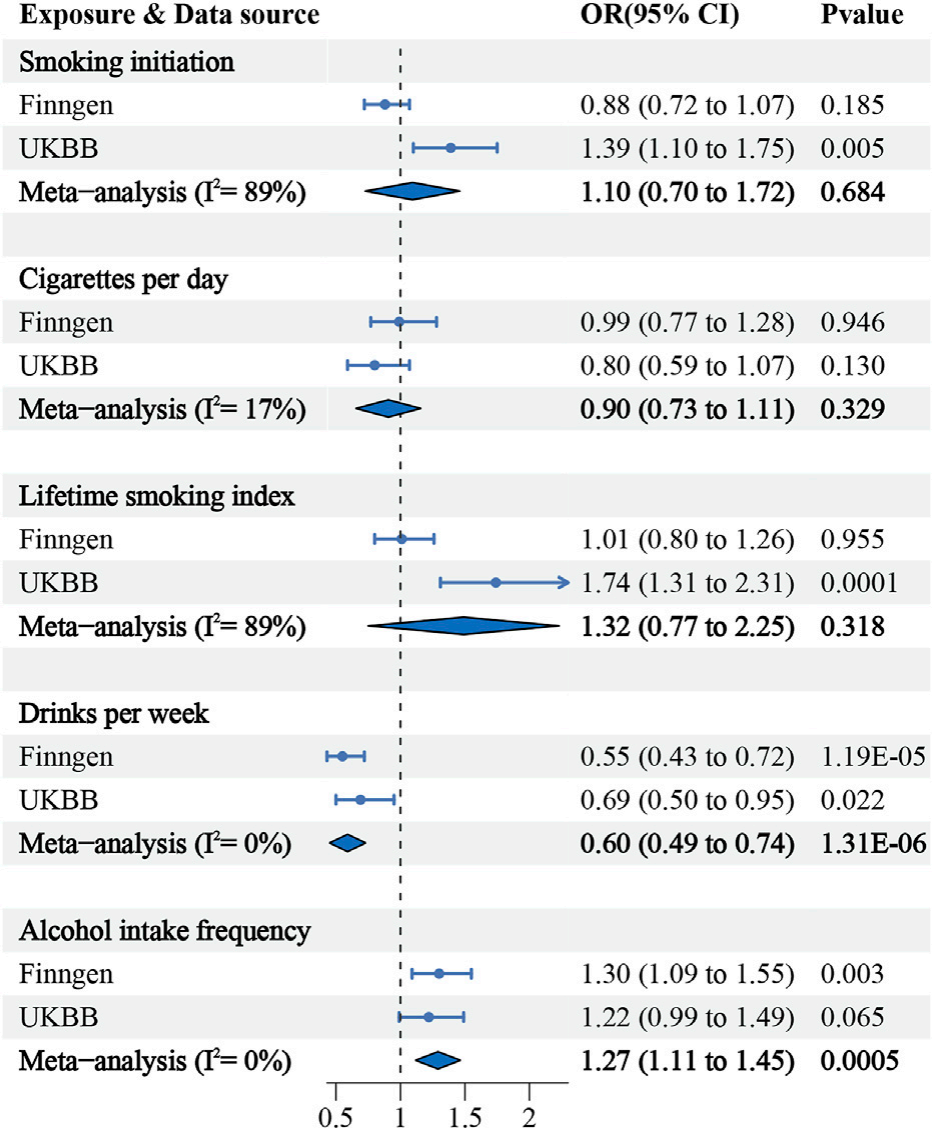
Association of smoking, alcohol consumption with the risk of upper urinary calculi in the MR.

Genetic predisposition to increased drinks per week was found to be significantly associated with a decreased risk of upper urinary calculi in both the FinnGen consortium and United Kingdom Biobank study. The combined OR for upper urinary calculi was 0.6 (IVW 95% CI: 0.49–0.74; *p* = 1.31 × 10^−06^) per standard deviation decrease in drinks per week. Interestingly, the genetically predicted alcohol intake frequency was associated with a significantly increased risk upper urinary calculi (IVW combined OR = 1.27; 95% CI: 1.11–1.45; *p* = 0.0005). Our study found no association between smoking initiation, the number of cigarettes per day, and the lifetime smoking index and the risk of upper urinary tract stones (Figure 2). These results were consistently reproduced in supplementary analyses (Supplementary Figure S1).

### 3.4 Multivariate MR analysis

**FIGURE 3:**
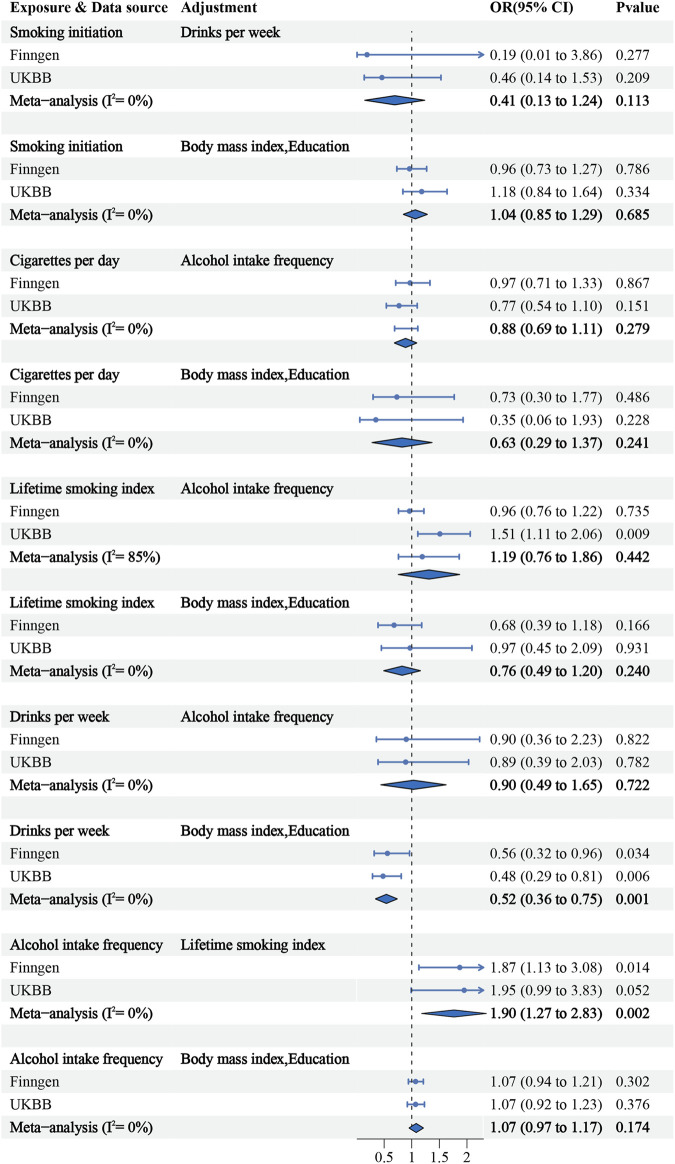
Forest plot for MVMR adjusted risk factors.

The PhenoScanner search identified links between IVs and traits related to education and obesity. After conducting multivariate IVW analyses, considering several potentially relevant adjustments including education and BMI, we observed that drinks per week remained significantly associated with a lower risk of upper urinary calculi (IVW combined OR = 0.52; 95% CI: 0.36–0.75; *p* = 0.001). However, the association between alcohol intake frequency and upper urinary calculi became non-significant (IVW combined OR = 1.07; 95% CI: 0.97–1.17; *p* = 0.174). Furthermore, when adjusting for the lifetime smoking index, alcohol intake frequency was found to be associated with a higher risk of upper urinary calculi (IVW combined OR = 1.90; 95% CI: 1.27–2.83; *p* = 0.002) (Figure 3).

## 4 Discussion

Using two-sample MR, we assessed for the first time the potential causal association between smoking and alcohol consumption on upper urinary calculi, and the results revealed that drinks per week was negatively associated with the occurrence of upper urinary calculi. This finding, together with other indicators, deserves in-depth discussion.

The effect of smoking on kidney stones is a subject of controversy. On one hand, Hamano et al. (Hamano et al., 2005) conducted a multivariate logistic regression analysis, revealing that smoking significantly increases the risk of kidney stones. Additionally, Liu et al. (Liu et al., 2009) identified smoking as an independent risk factor for the development of calcinuria. Notably, Soueidan et al. (Soueidan et al., 2015) conducted an investigative study, which indicated that patients with kidney stones had a higher prevalence of smoking (7% vs 21%, *p* = 0.02), and they were 8.5 times more likely to be current smokers. Potential mechanisms through which smoking contributes to urolithiasis formation include a considerable increase in plasma antidiuretic hormone due to smoking, leading to reduced urine output and promoting urinary supersaturation of crystals. Additionally, smoking contributes, to a lesser extent, to an increase in the production of reactive oxygen species (ROS), As signaling molecules and involved in receptor regulation, ROS activate transcription factors via P38 mitogen-activated protein kinase (-MAPK)/JNK. Thus, ROS-induced transcriptional activation leads to the production of prostaglandins and pro-inflammatory factors that impair endothelial function (Coe et al., 2005; Liu et al., 2009; Wigner et al., 2021). On the other hand, two cross-sectional studies conducted on Chinese populations found no significant association between smoking and stone formation (Dai et al., 2013; Zhao et al., 2015). Moreover, Słojewski et al. (Słojewski et al., 2009) reported an increase in urinary Hg levels in smokers, but the statistical significance was moderate. This finding does not support a possible association between smoking and urinary tract stone formation.

Currently, there is a divergence of opinions regarding the impact of drinking alcohol on the formation of urinary stones. According to Siener and others. The study discovered that individuals who consumed alcohol had higher levels of calcium in their urine, leading to temporary hypercalciuria. This could potentially raise the risk of developing calcium oxalate stones (Siener, 2006). Moreover, it is believed that alcohol enhances the production of uric acid and elevates the likelihood of developing uric acid stones. Furthermore, the consumption of alcohol causes oxidative stress on the tissue of the kidneys, which may potentially facilitate the development of kidney stones (Jones et al., 2021). It is crucial to emphasize that certain research has indicated a possible safeguarding impact of drinking alcohol. An analysis of multiple studies found that drinking alcohol was linked to a decreased overall chance of developing urolithiasis (OR = 0.683, 95%Cl: 0.577–0.808). Moreover, a correlation was observed suggesting that for each 10 g/day rise in alcohol consumption, there was a corresponding 10 percent decrease in the occurrence of urolithiasis (Wang et al., 2015).

Alcohol, on the other hand, hinders the release of vasopressin, resulting in an augmentation of urine output and dilution of urine. However, beer might also include defensive compounds discovered in hops. Studies have demonstrated that xanthohumol and humulone, which are the key components found in hops extract, possess potent abilities to suppress bone resorption (Tobe et al., 1997). As a result, these substances decelerate the release of calcium from the skeletal system and decrease the excretion of calcium. Lastly, red wine contains antioxidants that act as inhibitors of stone formation (Zecher et al., 2009). Indeed, considering the permanent harm caused by substances like acetaldehyde when alcohol is ingested beyond the limit, it is imperative to have a thorough and precise comprehension of alcohol intake.

### 4.1 Strength and limitation

Our MR analysis has the following advantages. Firstly, this is the first large-scale use of GWAS data to infer causal associations between smoking, alcohol consumption and upper urinary calculi, thereby reducing confounders and reverse bias. Secondly, the study population included only individuals of European origin, minimizing population stratification interference. Finally, sensitivity analyses and different model estimations were used to ensure the reliability of the results.

However, certain limitations are unavoidable. Firstly, the results of the study failed to be validated in other populations. Moreover, smoking and drinking habits are often combined, making it difficult to analyze them as independent factor. Secondly, there was a lack of data on alcohol type and consumption levels. Also, there was a failure to differentiate comparisons between genders, and multivariate analyses were unable to overcome bias because of multiplicity of effects in pathways other than education or obesity. Finally, future studies require larger sample sizes and precise stratified analyses to identify underlying physiopathological mechanisms.

## 5 Conclusion

There may be a causal link between drinks per week and risk of upper urinary calculi in people of European ancestry. In contrast, the detrimental effect of alcohol intake frequency on upper urinary calculi became non-significant after adjusting for BMI and education, and there is a need to further validate the potential effects and mechanisms of action of appropriate alcohol consumption on urolithiasis in the future.

## Data Availability

The datasets presented in this study can be found in online repositories. The names of the repository/repositories and accession number(s) can be found in the article/Supplementary Material.
