# Supplementary figures and images for: The causal association between smoking, alcohol consumption and risk of upper urinary calculi: insights from a Mendelian randomization study
Source: Front Genet. 2023 Nov 30;14:1268720. doi: 10.3389/fgene.2023.1268720 (PMC10723958; doi:10.3389/fgene.2023.1268720)

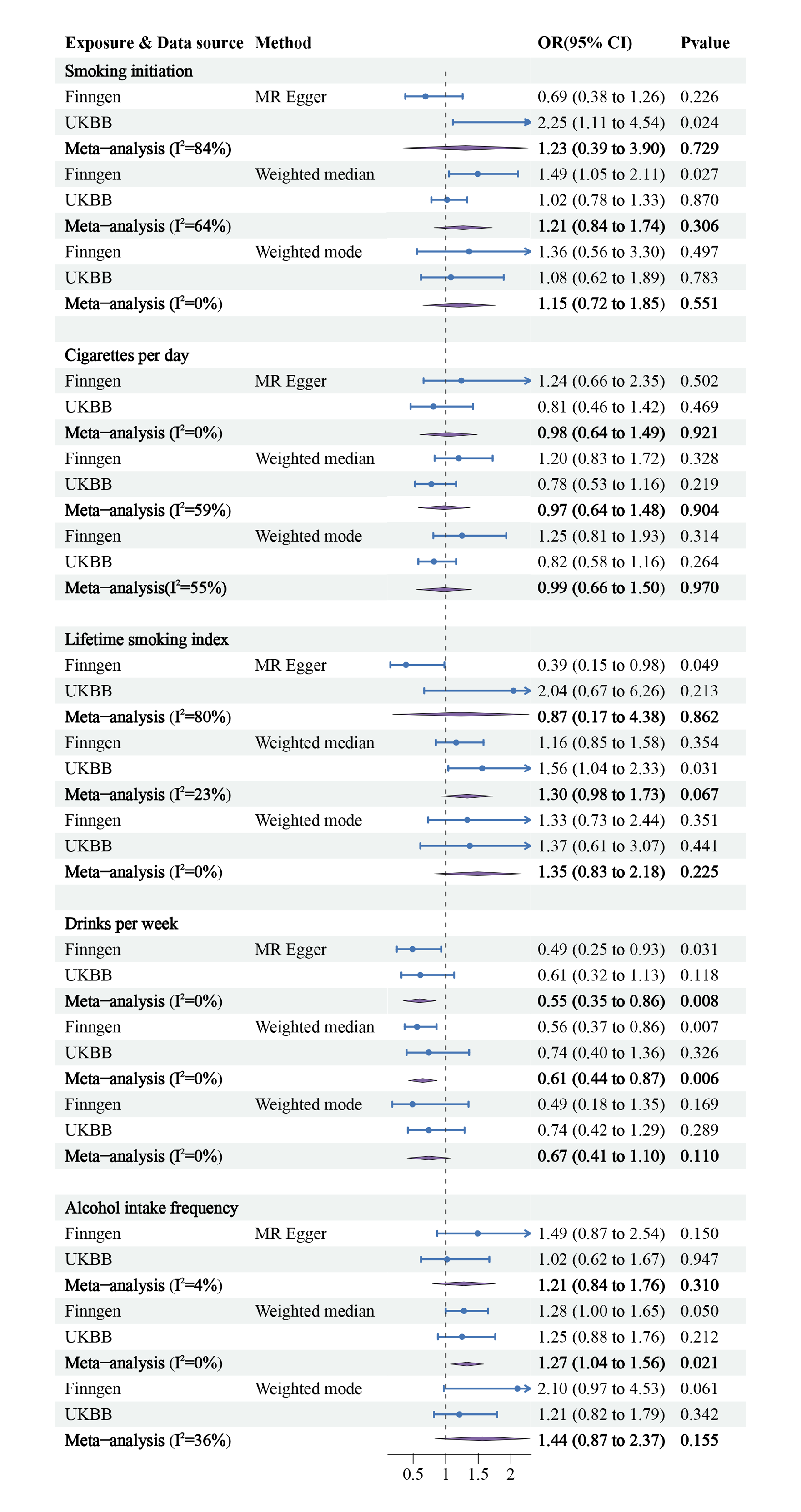

Supplement: Supplementary file 1 [file Image1.TIF]
